# Brothers in Arms: Th17 and Treg Responses in *Candida albicans* Immunity

**DOI:** 10.1371/journal.ppat.1004456

**Published:** 2014-12-04

**Authors:** Natasha Whibley, Sarah L. Gaffen

**Affiliations:** Division of Rheumatology & Clinical Immunology, Dept. of Medicine, University of Pittsburgh, Pittsburgh, Pennsylvania, United States of America; Duke University Medical Center, United States of America

## Introduction

Following the discovery of T helper 17 (Th17) cells in 2005, considerable research efforts identified interleukin 17 (IL-17) and Th17 responses as essential components of immunity to the commensal fungus *Candida albicans*. Much less is understood about regulatory T cells (Tregs) in candidiasis. However, emerging data point towards a surprisingly complex relationship between IL-17/Th17 and Treg responses during *C. albicans* infections, wherein Tregs both suppress and enhance immunity. This review will discuss the role of these responses during candidiasis and the consequences for disease outcome and therapy.

## IL-17/Th17 Responses Are Key Mediators of *C. albicans* Antifungal Immunity

IL-17-mediated immunity is crucial for protection against *C. albicans* infections, especially mucocutaneous infections, including oral and dermal candidiasis (reviewed in [1]). “Experiments of nature” have revealed mutations in humans that cause susceptibility to chronic mucocutaneous candidiasis (CMC), nearly all of which impact the IL-17/Th17 pathway (Table 1, reviewed in [2]). For example, individuals with mutations in *IL17RA*, *IL17RC, IL-17F*, or the IL-17 family–specific signaling molecule *ACT1* suffer from CMC [3], [4] (Casanova and Puel, personal communication; see Acknowledgments). CMC can be defined as a heterogeneous group of disorders characterized by persistent or recurrent *Candida* infection of mucosal membranes, skin, and nails. To date, there is no animal model that fully recapitulates the complex phenotype of CMC. However, models of oral and dermal candidiasis are in agreement with human data. IL-23^-/-^, IL-17RA^-/-^, IL-17RC^-/-^, and Act1^-/-^ mice are susceptible to oropharyngeal candidiasis (OPC) [5]–[7]. Similarly, IL-23^-/-^ and IL-17A^-/-^ mice display susceptibility to dermal candidiasis [8]. Somewhat surprisingly, IL-17RA^-/-^ and IL-23^-/-^ mice are not susceptible to vaginal candidiasis [9]. Although one study demonstrated that pharmacological blockade of Th17 responses increased vaginal fungal burdens, that study did not measure markers of symptomatic infection [10]. Therefore, IL-17-mediated immunity in candidiasis appears to be site dependent, though the underlying basis for this tissue specificity is enigmatic.

**Table 1:** Human genetic defects associated with susceptibility to *Candida* infections.

Gene	Mutation	Disease Phenotype	Reference
DECTIN1	Autosomal recessive	CMC	[51]
CARD9	Autosomal recessive	CMC and disseminated infection	[15]
STAT3	Autosomal dominant	Hyper IgE Syndrome	[52], [53]
TYK2	Autosomal recessive	Hyper IgE Syndrome	[54]
DOCK8	Autosomal recessive	Hyper IgE Syndrome	[55]
IL17RA	Autosomal recessive	CMC	[3]
IL17F	Autosomal dominant	CMC	[3]
ACT1	Autosomal recessive	CMC	[4]
STAT1	Autosomal dominant	CMC	[56], [57]
IL12RB1	Autosomal recessive	CMC	[58]
AIRE	Autosomal recessive	APECED/APS1	[59], [60]
CD25	Autosomal recessive	Oral and esophageal candidiasis	[45]
IL17RC	Autosomal recessive	CMC	Casanova and Puel, personal communication

Adapted from references [1], [2] and Casanova and Puel (personal communication). Abbreviations: APECED, autoimmune polyendocrinopathy-candidiasis-ectodermal dystrophy; APS1, autoimmune polyendocrine syndrome type 1; IgE, immunoglobulin E.

*C. albicans* also causes disseminated infections, associated with mortality rates of 50% or higher [11]. IL-17RA^-/-^ and IL-17A^-/-^ mice show elevated susceptibility to disseminated candidiasis [12]–[14]. However, humans with mutations in the IL-17 pathway typically do not develop disseminated disease. One exception is patients with *CARD9* mutations, who display susceptibility to both CMC and disseminated infection [15]. Why other IL-17 pathway gene mutations do not predispose patients to heightened susceptibility to disseminated candidiasis is unknown, although the number of patients identified with such mutations is limited. It is possible that under predisposing conditions (antibiotic treatment, intravenous catheter use, or abdominal surgery), individuals with impairments in the IL-17 pathway may be at increased risk for disseminated candidiasis, an issue that will need to be monitored, particularly considering the impending use of anti-IL-17 biologic therapy for autoimmunity [16].

## IL-17 Function and Sources

IL-17 exerts protective effects principally through the recruitment and activation of neutrophils. IL-17 primarily acts upon nonhematopoietic cells by stimulating the production of cytokines and chemokines, such as granulocyte-colony stimulating factor (G-CSF), interleukin 8 (IL-8) (humans), CXCL1, and CXCL5, which serve to expand and recruit neutrophils [1]. Depletion of neutrophils renders mice susceptible to OPC [17] and disseminated candidiasis [18]. Additionally, IL-17 signaling promotes anti-*Candida* killing mechanisms such as production of antimicrobial peptides (e.g., salivary histatins, β-defensins, and S100A8/9) [5], [9], [19].

CD4^+^ T cells are traditionally considered to be the primary cellular source of IL-17 during mucosal *C. albicans* infections [5], [20]. This assumption is based on the observation that patients with HIV/AIDS exhibit dramatically heightened susceptibility to OPC [21]. Moreover, most *Candida*-specific memory T cells in humans are Th17 cells. Similarly, in models of adaptive immunity, Th17 and not Th1 cells are induced by *Candida* and are protective against oral infections [20], [22].

IL-17 is produced by both conventional Th17 cells and by innate cells [23]. One recent report proposed a role for innate lymphoid cell (ILC) production of IL-17 in host defense against OPC [24]. However, IL-17 production by ILCs was not directly demonstrated. Notably, Rag1^-/-^ mice, which lack T cells but have enriched numbers of ILCs, are highly susceptible to OPC [20], [25], raising questions about the relevance of ILCs in oral candidiasis. Our recent data show that following immediate exposure to *C. albicans*, oral IL-17 is produced not by ILCs but by γδ-T cells and a subset of CD4^+^TCRβ^+^ innate-like cells known as “natural” Th17 cells [26]. Whether one or all of these IL-17^+^ subsets are necessary for host defense in humans remains to be determined.

## Treg Cells: Regulators of Infectious Disease

Tregs are a distinct subset of CD4^+^ T cell whose primary function is to restrict potentially pathogenic inflammatory immune responses. Tregs possess an extensive armory of suppressive mechanisms that can be cell contact dependent (acting through inhibitory receptors such as cytotoxic T-lymphocyte-associated protein 4 [CTLA-4]) or cell contact independent (acting via inhibitory cytokines and generation of suppressive metabolites) (reviewed in [27]). This suppressive tool kit makes Tregs adept at controlling cell types from both the innate and adaptive arms of the immune system. Several Treg subsets exist, including Tregs expressing CD25 and the canonical Treg transcription factor Foxp3 that will be the focus of this review. Foxp3^+^ Tregs can be further divided into thymus-derived Tregs, which are fully differentiated in the thymus, and peripherally derived (p)Tregs, which differentiate from naïve CD4^+^ T cells in the periphery following antigen stimulation. Although a detailed description of Treg biology is beyond the scope of this review, we refer the reader to several excellent reviews for further information on this subject [27], [28].

It is now appreciated that Tregs contribute to immunity against infectious pathogens. Inflammatory effector responses are critical in host defense against pathogens. However, excessive inflammatory responses can be damaging and therefore must be tightly regulated. A beneficial role for Treg-mediated restraint of immunopathology has been demonstrated in several viral and parasitic infections [29], [30]. In some settings, Tregs are also required for long-term maintenance of protective immunity, for example, in the context of *Leishmania major* infection [31]. Conversely, overly potent Treg suppression can inhibit protective immunity, favoring the pathogen. A detrimental role for Treg suppression has been demonstrated during *Mycobacterium tuberculosis* infection, in which depletion of Tregs resulted in enhanced protective responses [32]. Tregs can also promote, rather than prevent, inflammation. During mucosal herpes simplex virus infections, Tregs promoted protective effector responses via immune cell recruitment to sites of infection [33]. Therefore, Tregs can have diverse impacts, depending on the infection.

## IL-17/Th17 and Treg Responses Are Intricately Linked during Candidiasis

Treg responses are elevated during *C. albicans* infections, suggesting a functional role. An increase in the proportion of CD4^+^CD25^+^ cells and expression of Foxp3 was detected in the mesenteric lymph nodes (LNs) and stomachs of mice intragastrically inoculated with *C. albicans* [34], [35]. Similarly, CD4^+^CD25^+^Foxp3^+^ cells expanded in mice systemically infected with *C. albicans* [36]. However, Treg-mediated responses to *C. albicans*, and indeed to other fungi, remain poorly understood.

**Figure 1:**
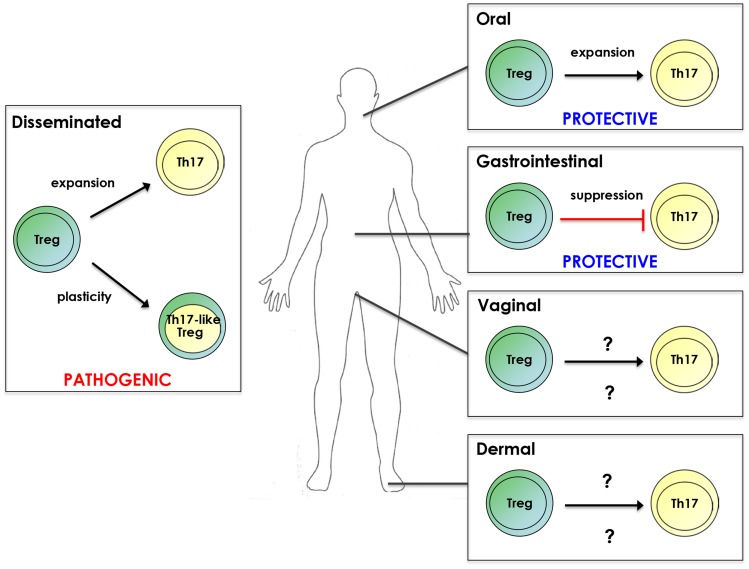
Treg/Th17 relationship during candidiasis. Tregs promote Th17 responses as well as acquire Th17 characteristics during disseminated infection. However, Treg/Th17 responses are associated with pathogenicity in this form of candidiasis. Conversely, Treg enhancement of Th17 responses during OPC is protective. Tregs suppress Th17 responses during gastrointestinal candidiasis, leading to decreased *C. albicans* colonization. Whether Tregs impact Th17 responses during vaginal and cutaneous candidiasis and the resulting outcome of disease remains to be determined.

Th17 and Treg subsets are reciprocally regulated during naïve T cell differentiation [37]. Reciprocal regulation of such responses was observed in a model of gastrointestinal candidiasis, in which increased Treg responses were associated with reduced Th17 responses and vice versa [34], [35]. Conversely, Tregs can also promote Th17 responses [37]. Accordingly, IL-17/Th17 and Treg responses are positively associated during OPC and disseminated candidiasis (Fig. 1). Treg depletion by anti-CD25 treatment results in concurrent depletion of Th17 cells during OPC. In the same model, co-transfer of CD4^+^CD25^+^ and CD4^+^CD25^-^ cells into Rag1^-/-^ mice enhanced protective Th17 responses [25]. In disseminated candidiasis, Tregs suppressed Th1 and Th2 responses while promoting Th17 responses in vitro [36]. Furthermore, Foxp3^+^ cell depletion in vivo was associated with reduced IL-17/Th17 responses [36]. Notably, both studies provide evidence that the mechanism of action is, at least in part, through consumption of IL-2 by Tregs through the high affinity IL-2R [25], [36]. IL-2 is essential for Treg survival but limits Th17 differentiation [38]. Therefore, Treg consumption of IL-2 reduces its local concentration, favoring Th17 development [25], [36], [39]. Whether IL-2 consumption by Tregs is a dominant mechanism for driving IL-17 responses during candidiasis remains an open question.

Plasticity is a phenomenon whereby CD4^+^ T cell subsets acquire characteristics of other populations (reviewed in [40]). For example, in some settings Tregs can express RORγt and produce IL-17A. Indeed, pTregs and Th17 cells possess an especially high degree of phenotypic flexibility [40], which has been observed in antifungal immunity. Specifically, dendritic cell recognition of β-glucans in the *Candida* cell wall by dectin-1 promotes conversion of Tregs to a RORγt^+^IL-17A^+^ phenotype [41]. Moreover, CD4^+^CD25^+^Foxp3^+^ cells isolated from systemically infected mice expressed RORγt and produced IL-17A, with the majority also expressing pTreg markers [36]. Collectively, these studies indicate that Tregs can promote IL-17/Th17 responses and acquire characteristics of Th17 cells in response to *C. albicans*.

## Final Outcome: Location, Location, Location

Although IL-17/Th17 and Treg responses can act cooperatively during candidiasis, disease outcome is strikingly different depending on infection site. In OPC, Th17 enhancement by Tregs increased resistance to infection [25]. In contrast, Treg enhancement of Th17 responses in disseminated candidiasis was associated with reduced resistance [36]. These studies suggest that inflammatory Th17 and Treg responses are protective at mucosal surfaces but pathogenic in systemic candidiasis. Consistent with this idea, humans with defective Th17 and Treg responses are susceptible to CMC but not to disseminated candidiasis [42]–[45]. However, the concept that elevated Th17 and Treg responses are harmful in disseminated candidiasis seemingly contrasts with the apparent protective role of IL-17 in mice [12]–[14], [36]. One explanation is that these studies use knockout animals with complete genetic ablation of IL-17 components and therefore do not address the requirement of balanced immune responses. In support of a pathogenic role for unbalanced Th17 and Treg responses during candidiasis, cytokines associated with Th17 responses positively correlate with increasing disease severity in disseminated candidiasis [46]. Similarly, overzealous Th17 responses are associated with immunopathology in gastrointestinal candidiasis [47]. Furthermore, depletion of Tregs during disseminated candidiasis increases resistance to disease [48]. Ultimately, the balance between protective versus pathogenic immunity is crucial in determining disease outcome.

How immune responses are shaped depends on factors in the microenvironment. Commensal microbes ferment dietary fibers to short chain fatty acids (SCFAs) that favor tolerogenic Tregs [49]. Additionally, transforming growth factor beta (TGFβ) and retinoic acid, which are enriched at the intestinal mucosa, promote Tregs over Th17 responses [50]. Since *C. albicans* is a commensal of human mucosae, it is likely that these tissues have evolved tolerogenic mechanisms to live in harmony with this fungus. In contrast, internal organs are shielded from the external environment and typically lack high levels of SCFAs and retinoic acid. Therefore, inflammation induced during disseminated *C. albicans* infection is more likely to go unchecked compared to mucosal surfaces, resulting in collateral tissue damage. Overall, site-specific factors are pivotal in dictating the balance between protective and pathogenic Th17 and Treg responses.

## Concluding Remarks

It is clear that IL-17/Th17 and Treg cells have a complex relationship, exemplified during infections with *C. albicans*. Although Th17 and Treg responses appear to be reciprocally regulated in certain situations (e.g., gastrointestinal candidiasis), Tregs promote Th17 activities and even acquire phenotypic characteristics of Th17 cells in other settings (e.g., oral and disseminated candidiasis). Notably, the impact of Th17 and Treg responses on disease outcome is distinct in different forms of candidiasis, highlighting the importance of microenvironment in shaping overall immunity. Elucidating the factors that determine the balance between protective versus pathogenic Th17 and Treg responses during candidiasis will be an important future avenue of research. Ultimately, it may be possible to exploit this information in order to help tune appropriate responses in the context of candidiasis.
